# Early cortical facilitation for emotionally arousing targets during the attentional blink

**DOI:** 10.1186/1741-7007-4-23

**Published:** 2006-07-20

**Authors:** Andreas Keil, Niklas Ihssen, Sabine Heim

**Affiliations:** 1 University of Konstanz, Konstanz, Germany

## Abstract

**Background:**

The present study aimed to investigate the time course of electrocortical facilitation for affectively arousing written words during the so-called 'attentional blink' (AB) period in a rapid serial visual presentation (RSVP) task. The AB refers to a period of reduced awareness for second-target stimuli following a first target by an interval of about 200–500 ms. Pleasant, neutral, and unpleasant written verbs were used as second targets in an 8.6-Hz RSVP paradigm that contained affectively neutral words as distractors. Replicating and extending behavioral studies, we expected that emotional second targets would be associated with better identification accuracy and greater electrocortical activity, compared with neutral targets.

**Results:**

The steady-state visual evoked potential was recorded using 129 scalp electrodes. The time-varying energy at the presentation frequency of 8.6 Hz was extracted as a continuous measure of electrocortical activity related to the RSVP stream. Behavioral data showed that at an inter-target interval of 232 ms, the report for emotionally arousing (pleasant and unpleasant) words was more accurate than for neutral control words. This result was mirrored by the electrocortical response at posterior sensors, which showed rapid amplitude enhancement (120–270 ms after T2 onset) for pleasant and unpleasant targets specifically.

**Conclusion:**

The present data suggest that identification facilitation for emotionally arousing target words in the AB is related to rapid enhancement of sensory processing. Affectively arousing information is preferentially selected at the level of early perceptual analysis, leading to facilitation at later stages of processing, including consolidation in working memory and visual awareness.

## Background

Affective processes can be viewed as action dispositions, regulating and optimizing an individual's response to motivationally relevant stimuli [1]. From an evolutionary point of view, it has been suggested that affectively arousing stimuli are preferentially selected from the sensory input [e.g., [2,3]]. In line with that notion, many authors have suggested that significant stimuli capture attention, leading to better performance and more accurate reporting for affectively arousing versus non-arousing exemplars [4]. This is consistent with electrophysiological work showing that the amplitude of cortical responses is specifically enhanced for affectively arousing visual stimuli at relatively early stages of processing, i.e., around 120–170 ms post-stimulus [5,6]. On the level of overt behavior, Öhman et al [7] observed that, in visual search, pictures of feared stimuli (e.g., spiders or snakes) are detected more rapidly among neutral distractors than are non-feared elements. These authors interpreted their findings as evidence for a parallel search process that serves the rapid detection of significant events. The question arises whether such perceptual and attentional facilitation is specific to unpleasant content, or affects both pleasant and unpleasant, compared with affectively neutral stimuli. Indeed, several authors have reported that aversive/defensive processing is distinct from appetitive arousal [8]. In the present research, we addressed this issue by using pleasant, neutral and unpleasant stimuli, with emotional (i.e., pleasant and unpleasant) stimuli matched for self-reported emotional intensity. This procedure has been widely used in studies of affective picture viewing [e.g., [9]], and has been related to physiological and brain data [10].

In addition to affective content communicated by means of pictures displaying faces or scenes, the effects of emotional words have attracted interest as a model for emotional processing. The most widely used paradigm to examine the effects of affective word content has been the lexical decision task, which requires subjects to make a choice response, indicating if a letter string is or is not part of a language's lexicon, i.e., whether it is a word or not [11]. Using this design, most researchers have observed advantages in speed and accuracy for affectively arousing words compared with low-arousal control words [e.g., [12]].

One outstanding question in this literature pertains to the selective enhancement of affectively arousing information communicated via a visual verbal channel. In particular, the time course of this process is relevant to theoretical models of emotion. This question can be approached using the so-called 'attentional blink' (AB) design, which allows studying selection from a temporal stream. The AB is a period of reduced awareness occurring, for instance, during rapid serial visual presentation (RSVP), when two targets need to be processed [13]. Usually, a first target (T1) is followed by a second target (T2) occurring later in the RSVP stream, with processing of T1 being critical for the occurrence of the AB [14]. Manipulating the lag (defined as the temporal position of T2 relative to T1) between these targets, a characteristic relationship between lag and performance has been reliably observed: At high rates of visual presentation (e.g., at 6 Hz and higher), T2s presented in an interval between 180 and 500 ms after a given T1 (i.e., typically at lags 2 and 3) are reported less accurately. Despite low accuracy during the AB, there is evidence that T2 items are semantically processed to a certain degree [15]. Moreover, late, but not early event-related potential (ERP) components were reduced [16] in trials comprising unidentified T2s [17]. These findings have led researchers to assume a post-perceptual, working-memory related interference process, which impairs performance for T2s within the AB period. Using emotional-word stimuli, it has been demonstrated that affectively arousing T2s are impaired to a lesser degree, being associated with more accurate report than affectively neutral T2s [18,19]. In a study using rapid streams of visual verbs, Keil and Ihssen [20] reported that identification performance for T2s was massively impaired when T2 was presented during the AB interval. Participants performed better in this critical interval (lag 2), however, when identifying affectively arousing (i.e., pleasant and unpleasant) compared with neutral T2 words. To elucidate the nature of attentional effects underlying such emotional modulation of visual awareness, it is essential to examine the temporal dynamics of attention allocation during the AB. In the present work, we used the steady-state visual evoked potential (ssVEP) as a continuous measure of attentional allocation directed to the RSVP stream [cf., [21]].

Studies employing ssVEPs capitalize on the fact that the visual brain responds to rapid visual stimulation at a fixed rate (i.e., flickering of a stimulus at 5–6 cycles per second or greater) with a near-sinusoidal response at the same frequency [22]. This oscillatory signal can be extracted at the known frequency using time-frequency analysis techniques such as complex demodulation [23]. The amplitude of ssVEPs has been shown to vary with aspects of selective attention, typically showing enhancement when a subject is paying attention to a stimulus [24,25]. In addition, amplitude changes in time mirrored the temporal dynamics of behavioral accuracy in experimental tasks requiring shifts of attention [21,26]. In terms of emotional stimulus content, Keil et al [9] found higher ssVEP amplitude and accelerated phase for emotionally arousing, compared with neutral control pictures. These differences were most pronounced at central posterior and right parietotemporal recording sites. The magnetocortical counterpart of the ssVEP, the steady-state visual evoked magnetic field, also varied with the emotional intensity associated with visual stimuli [27]. To summarize, the amplitude of evoked oscillatory brain responses has been demonstrated to vary as a function of emotional arousal and has been interpreted to reflect automatic allocation of attentional resources to affective stimuli. As a time-varying measure, the ssVEP is capable of reflecting dynamic changes in electrocortical activation related to an external event.

**Figure 1:**
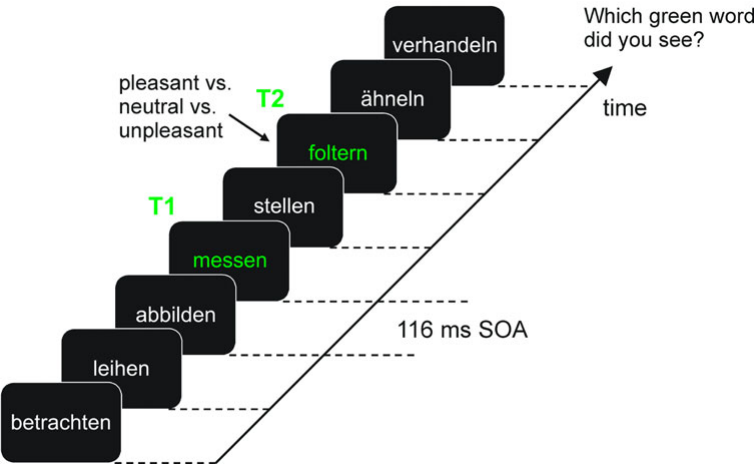
**Schematic of the experimental procedure**. Each experimental trial contained a baseline period of distractors, varying in number, before T1 was presented as the first verb, shown in green on the computer screen. RSVP was effected by using on/off duty cycles of 50 ms (word) and 66 ms (blank screen), resulting in 116 ms per cycle and a presentation rate of 8.6 Hz. The number of intervening distractors between T1 and T2 was manipulated to yield three lag conditions: lag 2 (one intervening distractor; see the present example), lag 4 (three intervening distractors), and lag 6 (five intervening distractors). Participants were asked to indicate which green words they had seen at the end of the trial, using a computer keyboard and a microphone. The example trial shown here illustrates a trial with lag 2, the first target being *"messen" *(to measure) and T2 being *"foltern" *(to torture).

The present study aimed to examine the time course of ssVEP amplitude changes in response to the inter-target interval and emotional content of second targets in an AB paradigm. To this end, we used the experimental design (see Figure 1) employed in a previous behavioral study [20]. We hypothesized that second targets having affectively arousing content would be associated with better identification accuracy during the AB period. Regarding electrocortical data, we expected amplitude enhancement for emotionally arousing T2s, paralleling the behavioral results. Given the electrophysiological evidence for an early enhancement in emotional perception, we expected that facilitation for emotional words would occur during initial processing of the T2 stimulus.

## Results

### Behavioral results

**Figure 2:**
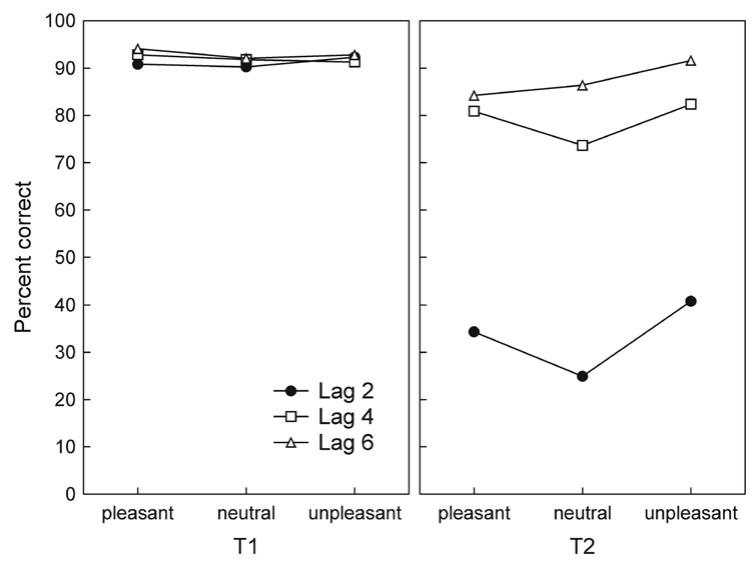
**Identification accuracy**. T1 performance (left panel) did not differ as a function of emotional content or lag. In contrast, T2 performance (expressed as percentage of correct trials, given that T1 was correctly identified in the same trial; right panel) showed sensitivity to lag and emotional content. Values reflect a mean of 13 participants.

As expected, accuracy of first target report was very high, participants averaging above 90% correct across all conditions. Figure 2 (left panel) illustrates that T1 report did not differ between experimental conditions. In contrast, analysis of variance (ANOVA) showed that the percentage of correct T2 responses varied as a function of lag (*F*(2,24) = 202.1, *p *< 0.0001), with accuracy gradually increasing from lag 2 to lag 6 (linear trend, *F*(1,12) = 293.6, *p *< 0.0001; see Figure 2, right panel). A main effect also emerged for emotional content (*F*(2,24) = 39.1, *p *< 0.0001), showing enhanced accuracy for pleasant and unpleasant, compared with neutral content, across lags. This pattern was characterized by a quadratic trend (*F*(1,12) = 43.2, *p *< 0.0001). Emotional content and lag interacted (*F*(4,48) = 3.0, *p *< 0.028), reflecting that identification of emotionally arousing content (pleasant and unpleasant) was superior to neutral content in the lag 2 condition (one intervening distractor) specifically (quadratic trend for lag 2, *F*(1,12) = 22.3, *p *< 0.001). This difference was less pronounced at lag 4 (*F*(1,12) = 9.2, *p *< 0.011), and absent at lag 6 (*F*(1,12) = 0.7, n.s.).

### Steady-state VEP amplitude: Effects of emotional content

**Figure 3:**
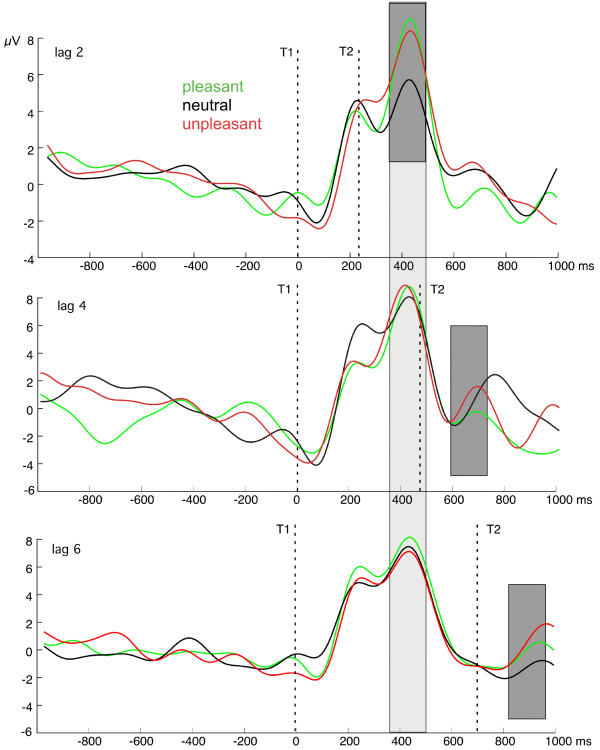
**Grand mean time-varying ssVEP amplitude**. Time course of the ssVEP amplitude at posterior amplitudes (see Figure 7) resulting from complex demodulation of the averaged EEG epochs for all lag by emotional content conditions (n = 13 participants). Amplitude shows facilitation in the lag 2 condition for emotionally arousing T2s, specifically. Fields shaded in gray denote the time ranges used for the statistical analyses. Light gray: comparison of amplitudes at fixed latency with respect to T1 (i.e., T1 locked, see Methods). Dark gray: comparison of amplitudes at fixed latency with respect to T2.

The time course of ssVEP amplitude for the nine experimental conditions is shown in Figure 3. This figure also illustrates the time windows used to examine amplitude changes relative to the onset of T1 and T2.

**Figure 4:**
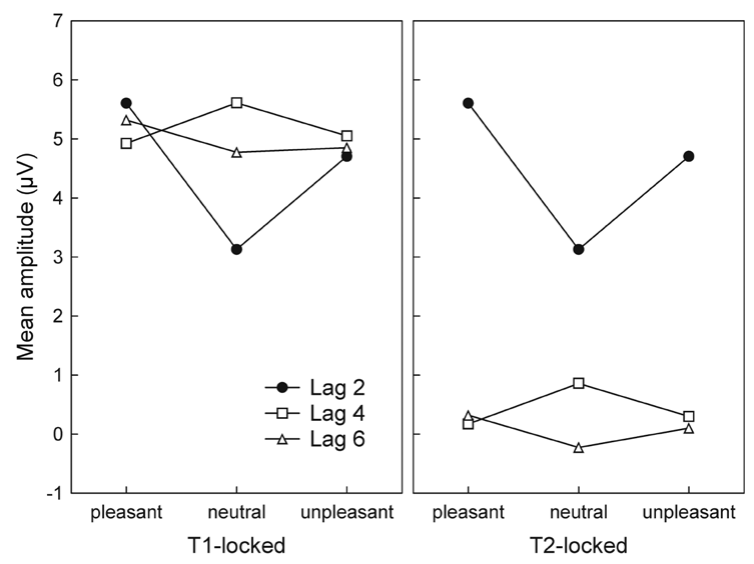
**Interaction plot for the ssVEP**. The time-varying amplitude in the time ranges of interest is as depicted in Figure 3. The left panel shows the analysis within a time range relative to T1. Amplitude is selectively decreased following neutral T2s in the lag 2 condition, suggesting interference by T2, which is not present for the arousing T2s (pleasant and unpleasant). Lag 4 and 6 conditions trivially do not exhibit modulation by content in this time range, as T2 occurs later in these conditions. They serve as comparison conditions, showing the enhancement related to T1 alone in the time range of interest. The right panel illustrates the mean amplitude across posterior electrodes at fixed intervals (120–270 ms) after T2 in each lag condition. Lag 2 is identical in both panels, as the post-T2 range was used as a reference for the T1-locked analysis.

**Figure 5:**
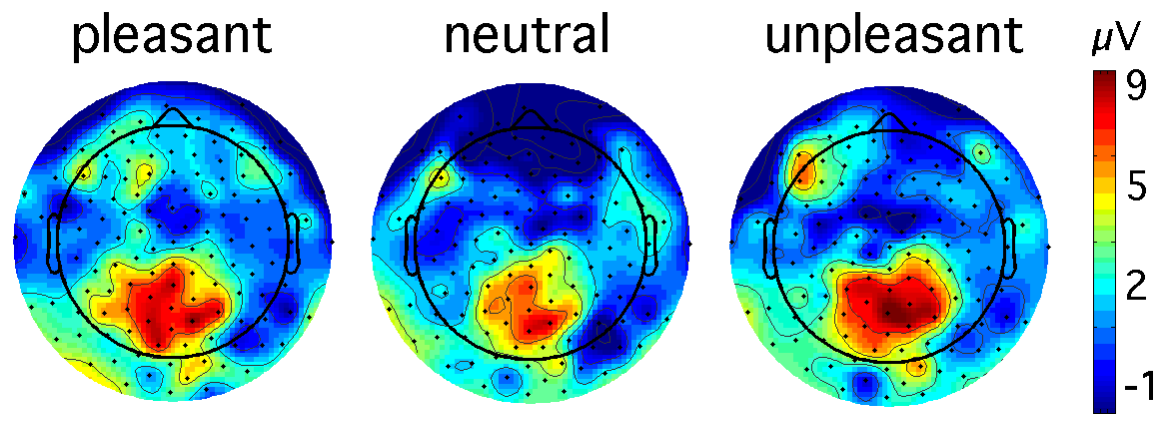
**Topographical maps: cortical response to T2 at lag 2**. Topographical distribution of time-varying amplitude, averaged in the time range between 120 and 270 ms, for the lag 2 condition and pleasant, neutral, and unpleasant T2 words. A grand mean across 13 participants is shown.

T2-locked analyses focused on a time window covering a time range between 120 and 270 ms following T2 onset, for each lag condition. We found a main effect of lag (*F*(2,24) = 7.1, *p *< 0.004), indicating greater amplitude at lag 2, compared with the other lags (linear trend, (*F*(1,12) = 7.6, *p *< 0.017). As can be derived from Figure 3, this result was due to the temporal proximity of T2 and T1 in the lag 2 condition. Mirroring the behavioral results, ssVEP amplitude following T2 onset exhibited sensitivity to emotional content for the lag 2 condition specifically. This difference is shown in Figure 4 (right panel) and yielded a significant lag-by-content interaction (*F*(4,48) = 3.3, *p *< 0.018). Quadratic trend analysis revealed that arousing content was associated with greater ssVEP amplitude at lag 2 solely (*F*(1,12) = 13.5, *p *< 0.003). The topographical distribution of the mean ssVEP amplitudes in the time windows of interest is depicted in Figure 5.

T1-locked analyses were conducted as a control, to follow up on the emotional effects in the lag 2 condition. In particular, we aimed to examine whether the affective modulation observed at lag 2 reflected an enhancement for arousing T2s or reduction for neutral T2s, compared with situations in which no T2 was presented. Examining a time window ranging from 352 to 502 ms following T1 onset across all lag conditions, we observed a lag × content interaction (*F*(4,48) = 3.3, *p *< 0.018; see Figure 4, left panel). At lag 2, ssVEP amplitudes to T2 onset matched the level of the amplitudes in the other lag conditions in which T2 occurred later than the window examined here. In contrast, neutral content in the early lag was specifically related to attenuated ssVEP amplitude. We tested this reduction for neutral T2s using multivariate contrast analysis, resulting in a Wilks lambda that reached significance (*F*(2,11) = 130.2, *p *< 0.0001).

### Correct versus incorrect trials

Complementing the analyses presented above, we also compared ssVEPs for lag 2 trials with correct and incorrect T2 report. Lag 2 was selected because it showed the central effect of this study behaviorally and at the neural level. We aimed to assess the similarity of electrocortical facilitation by emotional content versus correct identification (see Methods). Hence, we employed an ANOVA having the within-subjects factors of target type (T1, T2), hemisphere (left, right), and accuracy (correct, incorrect). Time windows following T1 and T2 were included in this analysis to examine a possible interaction of the temporal dynamics linked to T1 versus T2 identification.

**Figure 6:**
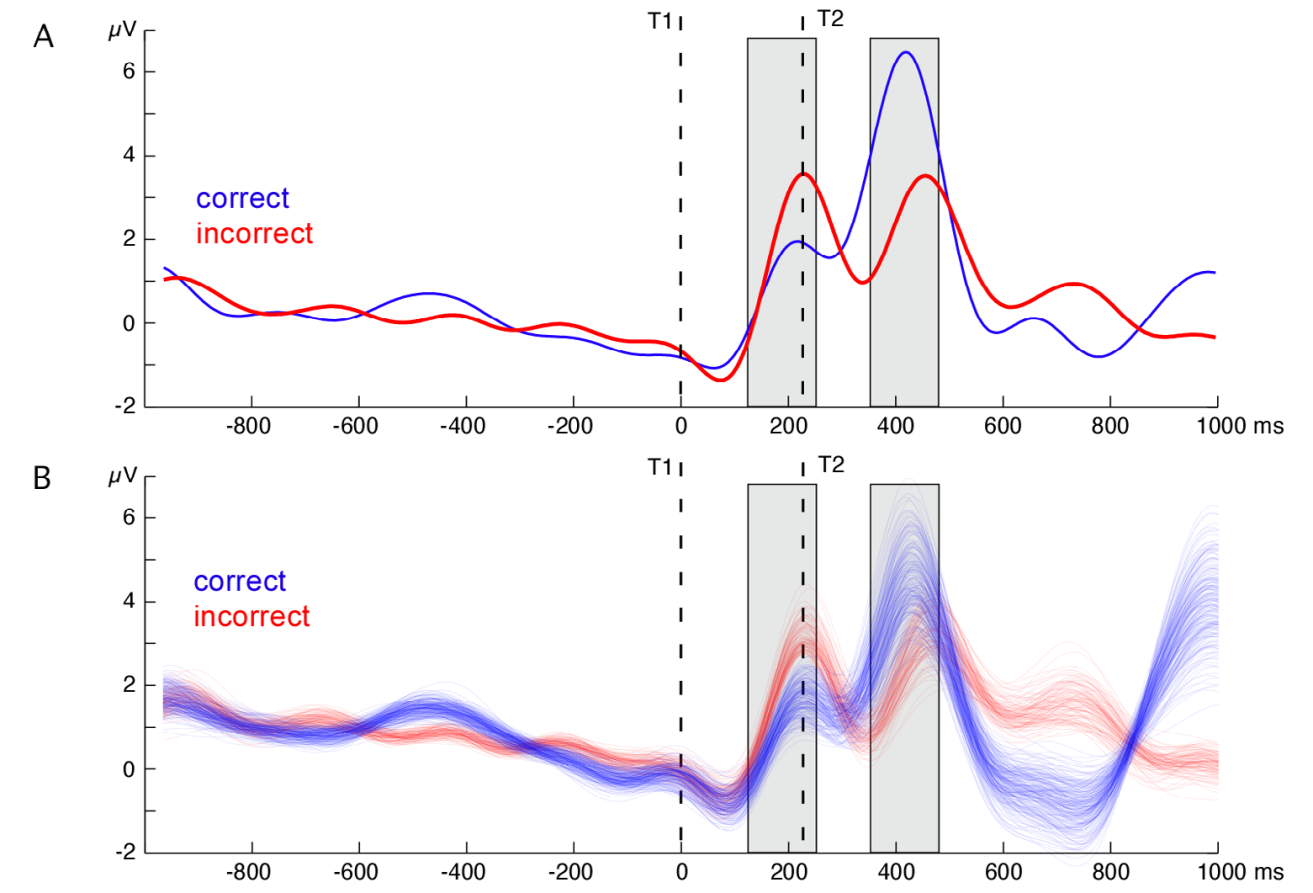
**Time course of ssVEP amplitude: correct vs. incorrect report**. **(A) **Grand mean time course of ssVEP amplitude across posterior electrode sites, for trials with correct versus incorrect report at lag 2, across emotion categories. Notably, trials with incorrect T2 response displayed a marked increase in amplitude following the T1, suggesting that greater cortical facilitation for the first target interferes with T2 identification. Response to T2 showed the opposite pattern, being associated with greater enhancement for correctly identified trials. **(B) **Grand mean time courses of ssVEP amplitude across the same electrode sites, for 500 grand means calculated from correct and incorrect T2 trials with arousing (pleasant and unpleasant) and neutral T2 trials equally contributing to the correct and incorrect condition, respectively. Different trials were sampled in a bootstrap approach from the entire set of trials on the level of individual participants, and grand means were generated 500 times after resampling for each individual. This analysis shows that early enhancement and late reduction of ssVEP amplitude for incorrect T2 trials is not due to unequal contribution of trials with emotion content to the correct/incorrect conditions (see Methods).

A main effect of target type (*F*(1,12) = 5.1, *p *< 0.043) indicated that the cortical response following T2 was greater overall than the response following T1. Further, correct trials showed reduced amplitude to T1 and enhanced activity in response to T2, compared with incorrect trials, which displayed the opposite pattern (target type × accuracy: *F*(1,12) = 8.5, *p *< 0.013; see Figure 6a). No effects of hemisphere were observed in any of the analyses. Because emotionally arousing (i.e., pleasant and unpleasant) targets were over-represented at lag 2 in the correct condition (and were under-represented in the incorrect condition), we performed an additional analysis using bootstrap resampling of trials at the level of individual participants (see Methods). Figure 6B shows the resulting grand mean traces reflecting 500 random draws from the available correct T1 trials for each participant, balancing the number of neutral and emotional T2s within individuals and conditions. We calculated a distribution of F values for the relevant target type × accuracy interaction, reflecting 500 resampling loops across participants. The resulting distribution showed that 99% of these *F*(1,12) values were greater than 6.9, which is well above the 5% level assumed for the present study (critical *F*(1,12) = 4.9).

## Discussion

The present study set out to examine the time course of electrocortical facilitation during identification of emotional word targets in an AB design. Replicating earlier work [20], we found that emotionally arousing words were less affected by the impairment typically associated with T1–T2 stimulus-onset asynchronies (SOAs) between 200 and 500 ms in RSVP streams. Emotionally arousing (i.e., pleasant and unpleasant) T2s were associated with enhanced identification accuracy by more than 10% in the lag 2 condition, which showed the greatest behavioral impairment. Behavioral findings were paralleled by modulations of ssVEP amplitude at latencies between 120 and 270 ms following T2 onset. In this time range, ssVEP amplitude over the posterior cortex showed specific facilitation for the emotionally arousing targets, but not for neutral targets during the lag 2 condition. Lag 2 was associated with generally enhanced amplitude compared with the lag 4 and lag 6 conditions. As can be seen from Figure 3, this enhancement was due to the time course of the electrocortical response following T1: Occurrence of the first target led to a pronounced amplitude increase that had a biphasic time course and lasted for about 500–600 ms, matching the duration of the AB period. The temporal pattern of the continuously measured electrocortical response thus suggests a strong role of the T1 for the subsequent perceptual events, as has been proposed in the majority of AB theories [e.g., [28]]. Facilitation for emotionally arousing T2 content was most pronounced at lag 2, for which the T2 occurred early enough to elicit secondary facilitation within the initial enhancement window. Comparative analysis of the amplitudes in that same time window relative to T1 indicated a selective reduction for the neutral T2s in the lag 2 condition; this attenuation did not affect emotionally arousing T2s.

The time-varying ssVEP amplitude as examined here has frequently been used in the context of attention-shifting studies, and has pointed to reflexive orienting towards task-relevant stimuli within 100 to 200 ms [29]. This coincides with the present finding of relatively early (120–270 ms) facilitation of electrocortical oscillations in response to emotional T2s. Hence, timing of modulations as observed here is consistent with studies reporting early involvement of visual analysis in lexicosemantic access. For instance, words having high versus low frequency in a given language are distinguished as early as 120–160 ms post-stimulus [30], which suggests parallel evaluation of different aspects related to visual words. Indeed, theoretical work has proposed a complex sequence of processing steps such as sublexical encoding of the visual language stimulus and subsequent lexical access [31]. It is conceivable that emotional content aids these subprocesses at various stages, facilitating word recognition [32] and initiating more efficient attention allocation to affectively arousing target words [11]. Recent research on emotional perception has emphasized that processes of attention allocation and facilitated perception may indeed be disentangled, and may have differential influence on visual analysis [33].

Given the low trial count in the lag 2 condition, it was not possible to analyze the effects of correct versus incorrect responses on the ssVEP for the full factorial design.

Thus, we examined trials with correct versus incorrect T2 report in lag 2 across affective T2 contents. This procedure aimed to shed light on the electrocortical correlates of correct/incorrect T2 identification at lag 2, and to compare them with effects of emotional content. We observed that trials with missed T2s were associated with relatively enhanced electrocortical responses to T1, which was identified correctly in all trials entering this analysis (see Methods). In contrast, trials with correct T2 identification showed reduced response to T1 and enhanced amplitude following T2. This enhancement following T2 was in the same time range as the facilitation by emotional content, i.e., occurring between 120 and 270 ms after T2 onset. This result may add to behavioral data showing that a decrease in volitional attention allocated to T1 may exert beneficial effects for T2 report [34].

The apparent similarity between the time courses of emotion facilitation and identification facilitation suggests that both successful T2 identification and affective content of T2 are associated with electrocortical facilitation at a relatively early perceptual level. Previous psychophysiological studies addressing the question of T2 awareness in AB designs have mostly relied on ERPs, which are obtained by time-domain averaging of electroencephalogram (EEG) segments [16]. Researchers have often focused on the P3 component of the ERP, which starts at about 300–400 ms post-stimulus, and has been regarded as a reliable and robust marker of higher-order processing. For instance, Kranczioch et al [17] found that electrocortical activity in the P3 range was attenuated for missed, and unsuppressed for detected T2 stimuli. Comparing the ERP time course for trials with and without T2 presentation in RSVP, as well as missed and identified targets, Sergent et al [35] observed: (i) no differences between seen and unseen T2s for early visual ERPs (i.e., P1 and N1); (ii) a linear decrease of the subsequent N2 component (around 270 ms post-T2) as a function of T2 visibility; and (iii) absence of later ERP components for unseen stimuli, with a threshold at about 50% probability of report. This pattern of results suggests that early ERP markers of visual selective attention remain unaffected by T2 visibility.

While the focus of the current work is on emotion facilitation, the ssVEP data presented here suggest that missed T2s are generally associated with a distinct pattern of temporal attention allocation: Augmented ssVEP amplitudes followed T1 onset and reduced amplitudes followed T2, compared with trials with identified T2 stimuli. Given the temporal uncertainty (here ≈120 ms) of time-frequency-analysis techniques, it is possible that the cortical facilitation extends over a period of 400 ms, ranging from early perceptual analysis well into the P3 window (300–400 ms after stimulus onset). Future work may investigate the spread of activation from initial sensory processing to higher-order stages, using methods with better spatial resolution. In fact, research using magnetoencephalography has pointed to the integrated activity of a specific set of brain structures (temporal, right parietal, and frontal cortex) mediating temporal attention during the AB, acting in phase synchrony at frequencies between 13 and 20 Hz, i.e., the beta range of the EEG [36]. Increased T1 activation during RSVP, as measured by means of magnetoencephalography, has also been related to inhibition of T2 report on the behavioral level, which is in line with the present results [37].

The present result of facilitated cortical processing of arousing T2s is also pertinent with respect to theories of the AB. Although recent studies [38,39] questioned the validity of serial bottleneck accounts, a majority of AB theories, e.g., the two-stage model by Chun and Potter [14] or the central interference model by Jolicoeur and Dell'Acqua [28], attribute the AB effect to a delay of T2 consolidation in working memory, which is elicited by T1 encoding. During the delay period, the T2 representation is fragile and thus can easily be overwritten by the trailing items [40]. According to this perspective, facilitated identification of arousing targets may arise because the initial representation of arousing T2s is less susceptible to distractor noise or to decay during the consolidation period. In a similar vein, so-called biased competition accounts of the AB [41] predict top-down modulation of the visual cortex by higher structures such as the frontotemporal cortex, protecting arousing targets during the AB. In particular, protecting the T1 against the masking effects of the following distractor may occur at the cost of speed, leading to problems with consolidating of rapidly following target items [42]. Although latency measures were not examined here, this perspective is in line with greater cortical activity following T1 in incorrect, compared with correct trials, possibly reflecting such a trade-off between T1 protection and T2 processing. Generally, these findings point to the relevance of T1 processing for subsequent T2 identification.

Electrocortical trade-off between T1 and T2 as a function of accuracy is also consistent with other theoretical accounts of the AB phenomenon. For instance, Potter et al [43] showed differential priming effects between two targets in an AB task, with T2 priming T1 at short SOAs. This finding is supportive of early competition between the representations of the targets, suggesting that T2 may be identified prior to T1, thus exerting a priming effect. In a two-stage competition model of report accuracy during rapid processing [44], our result of amplitude trade-off in early time ranges might be taken as evidence for competition between target stimuli at a first stage (identification). Recent attempts to describe the AB as a lapse of attentional control elicited by the T1 features [45] could also be accommodated with the present results. Although caution is warranted regarding the interpretation of time-varying ssVEP as reflecting solely attentional selection, the biphasic increase in electrocortical activity following T1 across conditions may indicate selection or filtering of visual features specific to T1. Future work including a lag1 condition within the present design may shed light on this issue.

When discussing emotional aspects of perception, memory, and action, one useful framework is the notion that affective experience and behavior may be described in terms of distributed networks [1]. In this model, perceptual networks representing affectively arousing stimuli are characterized by strong connections to memories, visceral systems, and motor systems that mediate executive functions, motor preparation, and action towards the affective stimulus, among other functions. During the course of competition with other stimulus representations, networks representing affectively arousing T2s may thus benefit from higher connectivity and larger extension of its connections.

## Conclusion

Our results strongly suggest that the affective content of words facilitates word processing during periods of reduced awareness. The processing steps modulated by emotional content may include early stages such as sublexical analysis, lexical access, and subsequent processing such as consolidation in working memory. Both electrocortical and behavioral facilitation effects related to emotional content were strongest in situations characterized by limited resources, i.e., the beneficial effects of word content appear to be most relevant when stimulus encoding and processing is made difficult by dual-task interference. We conclude that affectively arousing information is preferentially selected at the level of early perceptual and early lexical analysis, leading to facilitation at later stages of processing, including consolidation in working memory and visual awareness.

## Methods

### Participants

Informed consent was obtained from 13 healthy university students (six women, seven men; mean age 21.9 years, SD = 3.7) having normal or corrected vision. They were given class credits or a financial bonus of EUR7.50 for participation.

### Stimuli

Stimuli and procedures were identical as described in Keil and Ihssen [20]. Based on an ongoing rating study, we selected 90 German verbs (30 pleasant, neutral, and unpleasant exemplars, respectively) to be maximally different regarding their emotional valence and arousal in a two-dimensional affective (valence × arousal) space. Neutral verbs included stimuli such as *to accompany*, *to continue*, and *to install*. Pleasant (e.g., *to admire*, *to hug*, *to win*) and unpleasant verbs (e.g., *to steal*, *to destroy*, *to lie*) differed from neutral items both in terms of valence and arousal ratings on the nine-point scale of the Self-Assessment Manikin [46]. Pleasant (mean arousal rating 7.06; mean valence rating 7.87), neutral (2.61; 4.99), and unpleasant (7.62; 1.61) verbs served as T2 stimuli in the RSVP task. Thus, pleasant and unpleasant T2 verbs were selected so as not to differ with respect to emotional arousal/intensity. T1 verbs and distractor verbs were selected from a pool of neutral German verbs for which ratings were obtained separately. T2 verbs in the three affective categories were matched for number of letters, number of syllables, affixations, and word frequency in the German language using the CELEX database [47].

### Procedure

Participants entered a sound-attenuated, dimly lit chamber, and were seated comfortably, with the chin on a rest. Stimuli were presented on a computer screen with a retrace frequency of 60 Hz, located at 700 mm distance from the observer. Target words were shown in green, distractor items in white, both on a black background, using Helvetica font, 26-point. All words had a luminance of 8.6 cd/m^2 ^and subtended a vertical visual angle of 0.82°. A script written using the Experimental Run Time System (ERTS) software controlled the presentation and response registration.

The experimental session started with four practice trials to demonstrate the procedure and make sure that all individuals understood the task correctly. In total, there were 270 test trials, organized into two blocks. A schematic of an example trial is shown in Figure 1. Each trial contained the following series of events. First, a blank screen appeared for 1000 ms, then, a stream of verbs at a frequency of 8.6 Hz was displayed in the center of the screen. The 8.6-Hz RSVP was effected by alternating the presentation of a word for 50 ms, followed by a black screen for 66 ms. Following the initial black screen, a baseline RSVP of neutral distractor words was displayed, with durations varying randomly between 8 and 25 words (i.e., about 928–2900 ms). This baseline RSVP was followed by the T1 neutral verb (first target), a varying number of neutral distractors, and the T2 (second target), again followed by a varying number of distractors. T1–T2 intervals varied to contain one, three, or five intervening distractor verbs (i.e., lag 2, lag 4, lag 6). Accordingly, SOAs were 232 ms (lag 2), 464 ms (lag 4), and 696 ms (lag 6). Each affective verb occurred three times during the experimental session, in different SOAs, respectively. Thus, there were 30 trials for each combination of SOA and affective content (pleasant, neutral, and unpleasant). The order of verbs and conditions was randomized, with the constraints that: (i) immediate repetitions of trials belonging to the same condition could not occur, and (ii) that verbs were not repeated within blocks of 90 trials.

At the end of each trial, subjects were asked via a message on the computer screen to report aloud the green words using a microphone in the experimental chamber and to type the first letter of the words on a computer keyboard. Participants started the subsequent trial after completing the report, via keyboard feedback.

### Analysis of behavioral data

In order to be labeled as correct, responses at the end of the RSVP trial were required to reflect the temporal position (first and second target) in the RSVP stream. Furthermore, only trials with correct T1 reports were considered for determining T2 accuracy. Accuracy of identification was then expressed as the percentage of correct responses for each of the nine experimental conditions (3 SOAs × 3 affective categories). Subsequently, separate *F *values for T1 and T2 responses were calculated using ANOVAs having the within-subject factors of lag (3: lag 2 = 232 ms SOA, lag 4 = 464 ms SOA, lag 6 = 696 ms SOA) and content (3: pleasant, neutral, unpleasant). The hypothesis of a quadratic relationship between word category and response accuracy was tested for each lag by means of planned comparisons (trend analyses) modeling a quadratic contrast (pleasant = unpleasant > neutral).

### EEG recording and data reduction

An EEG was continuously recorded from 129 electrodes using an Electrical Geodesics™ (EGI) high-density EEG system and digitized at a rate of 250 Hz, using Cz as a recording reference. Impedances were kept below 50 kΩ, as recommended by the manufacturer. A subset of EGI net electrodes located at the outer canthi, and above and below the right eye, was used to determine horizontal and vertical electrooculogram (EOG). All channels were preprocessed online by means of 0.1 Hz high-pass and 80 Hz low-pass filtering. Epochs were extracted from the continuously recorded EEG relative to the onset of T1 for each stimulus, using 2800 ms pre-T1 and 2000 ms post-T1. The mean voltage of a 1000-ms segment preceding T1 onset was subtracted as the baseline. In the next step, data were low-pass filtered at a frequency of 40 Hz (24 dB/octave) and then submitted to the procedure proposed by Junghöfer et al [48]. This procedure uses statistical parameters of the data to exclude channels and trials that are contaminated with artifacts. Recording artifacts are first detected using the recording reference (i.e., Cz), and then global artifacts are detected using the average reference. Subsequently, distinct sensors from particular trials are removed based on the distribution of their amplitude, standard deviation and gradient. Data at eliminated electrodes are replaced with a statistically weighted spherical spline interpolation from the full channel set [48].

**Figure 7:**
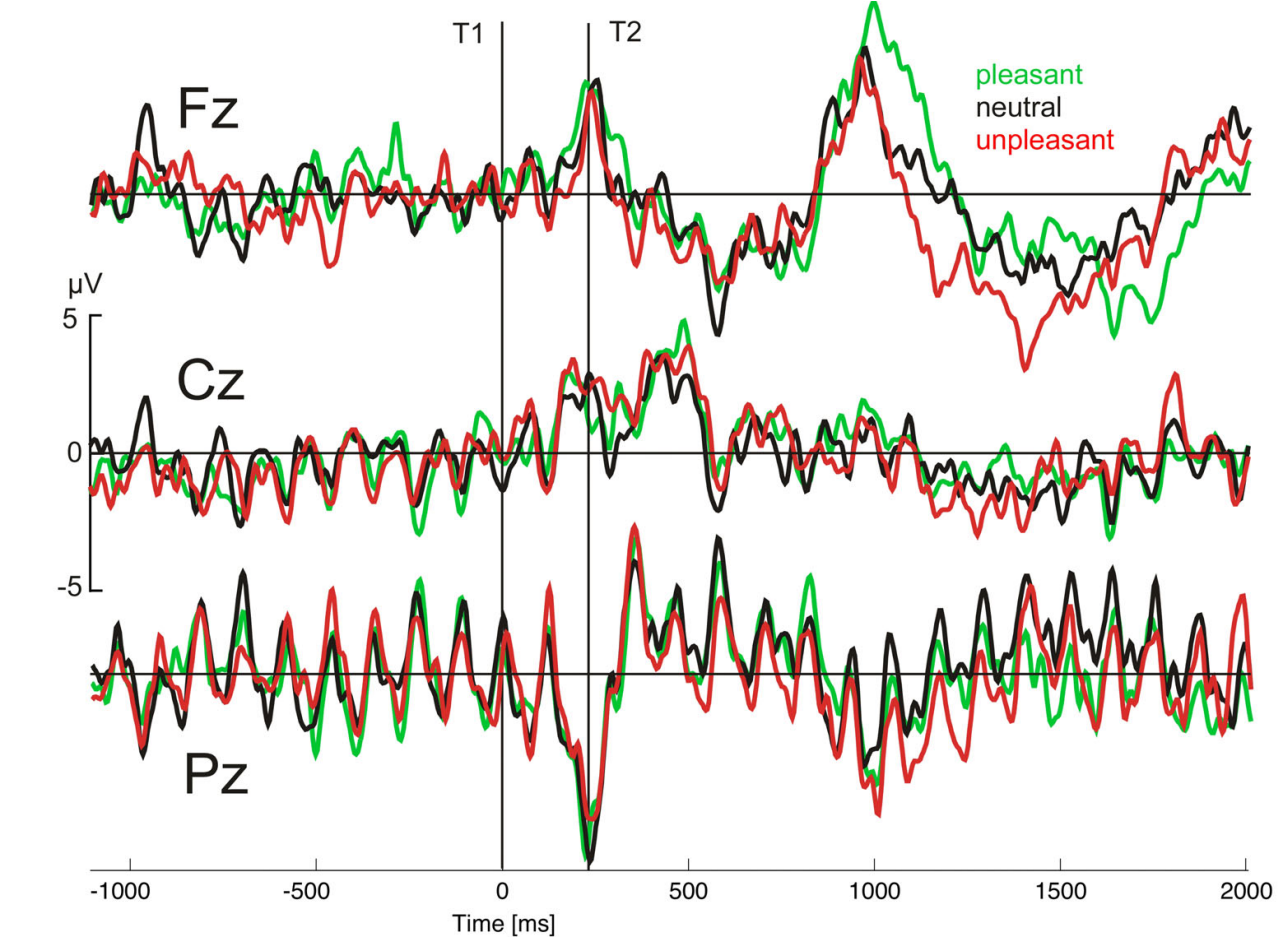
**ssVEP time series**. Grand mean ssVEP time series in the time domain, prior to complex demodulation, at three electrode sites roughly corresponding to Fz, Cz, and Pz of the international 10–20 system. Data for the lag 2 condition and pleasant, neutral, and unpleasant T2s are shown.

The mean number of approximated channels across conditions and subjects was 8.4. It was ensured that the rejected sensors were not located within one region of the scalp, because this would make interpolation for this area invalid. Spherical spline interpolation was used throughout both for approximation of sensors and illustration of ssVEP amplitude maps [49,50]. Single epochs with excessive eye movements and blinks or more than 14 channels containing artifacts were discarded. The resulting data were then visually inspected together with the vertical and horizontal EOG to exclude remaining artifacts. Subsequently, data were arithmetically transformed to the average reference, which was used for all analyses. After artifact correction, an average of 78% of the 270 trials was retained in the analyses, with lag or content conditions not being statistically different from each other. Epochs were then averaged according to the 3 × 3 design of the study, yielding ssVEP time series for the nine experimental conditions at 129 electrodes for each subject. Figure 7 shows the grand mean ssVEP time series after averaging for three selected electrode sites in the lag 2 condition. To examine effects of correct identification in the most difficult condition (lag 2), artifact-free epochs were averaged across affective content for correct versus incorrect trials. This procedure aimed to illustrate the electrocortical dynamics associated with successful versus non-successful identification. Separate analysis of correct/incorrect trials within the 3 × 3 design was not possible because of the low trial count in the lag 2 correct condition.

### Steady-state VEP analyses

Time-varying amplitude at the stimulation frequency of 8.6 Hz was extracted by means of complex demodulation [23]. The averaged data were multiplied with a sine and cosine function at the stimulation frequency. The resulting time series were then low-pass filtered at a cut-off of 2 Hz, leading to sensitivity of the resulting waveforms to amplitude changes between 6.6 Hz and 10.6 Hz, with a center frequency of 8.6 Hz. In the final step, sine and cosine time series were pooled as the vector length for each time point, i.e., the square root of the sum of the squares, resulting in time-varying amplitude at each sensor. The mean voltage of a segment extending from -1000 to -100 ms relative to T1 onset was subtracted as the baseline, and amplitude was thus expressed as change against baseline. Subsequently, three aspects of the time-varying amplitude were examined statistically.

(i) T2-locked analysis: In accordance with earlier work, we expected that effects of target processing on ssVEP amplitude recorded over the posterior cortex would occur within 1–2 cycles after the target event [21,29]. We therefore formed averages over time segments following T2 onset by 120–280 ms for each lag condition and emotional content.

(ii) T1-locked analysis: To compare affective modulation at lag 2 with the other lag conditions at a fixed temporal relationship to T1, we selected the same time range as used for the lag 2 analysis of T2 (i.e., 120–280 ms following T2 at lag 2, which corresponds to 352–502 ms after T1) for all lag conditions. Thus, the effects of presence of T1 alone versus T1 and T2 could be examined using these time averages.

(iii) Comparison of correct versus incorrect T2 trials: Given the low trial count for correct trials in the lag 2 condition, it was not possible to use a full factorial design together with a factor of correct-incorrect responses (see above). We therefore compared the ssVEP time course for trials with correct T1 and correct versus incorrect T2 report across emotional contents. Two time windows were formed for the ssVEP reflecting correct and incorrect T2 identification, following each target by 120–270 ms. Because T2 accuracy at lag 2 systematically varied as a function of emotional content, an additional bootstrap resampling procedure was performed to control for potential confounds of emotional content for the correct-incorrect analysis. This procedure included recalculating the condition (correct versus incorrect T2 given correct T1) averages for each participant with equal numbers of neutral and arousing (pleasant and unpleasant) trials. These trials were randomly drawn from the pool of available trials (with correct T1 response, varying in terms of T2 accuracy) in each participant. This method eliminated the bias in favor of trials with emotionally arousing T2s in the correct condition and *vice versa*, on an individual level. Repeating the randomization across all participants for 500 times allowed recalculation of the ANOVA design for each loop across participants, generating a distribution of *F *values that reflected the variation due to trial sampling. Subsequently, the lower 1% margin of this distribution was compared with the *F *distribution for the respective test statistic to assess statistical significance.

**Figure 8:**
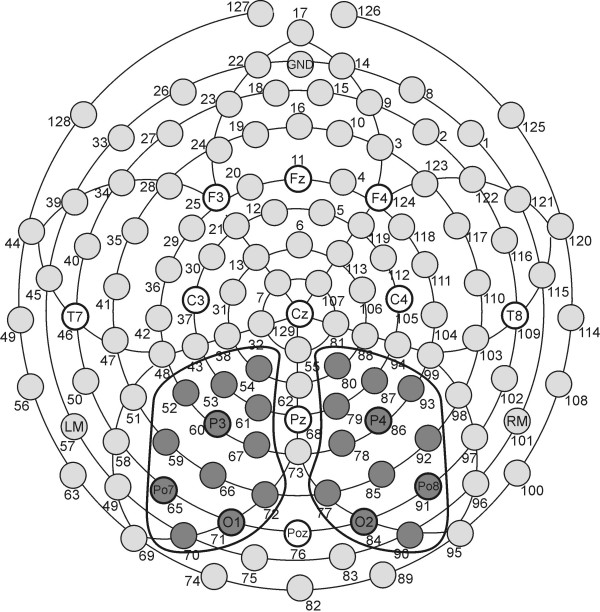
**Layout of the electrode array**. Top view of the electrode array used in the present study. Shaded electrode sites in the marked clusters were selected to form regional means entering statistical analyses.

All temporal averages were pooled for two sensor groups at parieto-occipital electrode sites, one in each hemisphere, where the ssVEP was most pronounced (see Figure 8). Paralleling behavioral data, mean ssVEP amplitudes were evaluated using ANOVA with a within-subjects factor of hemisphere in addition to the lag and emotional content factors. Again, significant interaction of lag and content were followed by contrast analysis for each lag, testing for a quadratic trend, with pleasant T2 = unpleasant T2 > neutral T2.

Electrocortical differences as a function of T2 accuracy at lag 2 were evaluated by means of ANOVA having within-subjects factors of hemisphere, target (post-T1, post-T2), and accuracy (correct, incorrect). Time windows and electrode groups were used as described above.

## Authors' contributions

AK conceived of the study, carried out the ssVEP data analyses and co-drafted the manuscript. NI designed the study, and contributed to data collection, data analysis, and manuscript drafting. SH participated in the data analysis and co-drafted the final version of the manuscript. All authors read and accepted the final version of the manuscript.
